# Picture Exchange Communication System (PECS) Implementation Program for children with autism spectrum disorder

**DOI:** 10.1590/2317-1782/20232021305en

**Published:** 2023-09-18

**Authors:** Ana Carina Tamanaha, Dayane Oliveira Felicio Olivatti, Simoni Camilo da Silva, Soraia Cunha Peixoto Vieira, Jacy Perissinoto

**Affiliations:** 1 Departamento de Fonoaudiologia, Universidade Federal de São Paulo - UNIFESP - São Paulo (SP), Brasil.; 2 Instituto de Educação de Londres - Belo Horizonte (MG), Brasil.

**Keywords:** Autism, Communication, Language, Communication Aids for Disabled, Speech Language Therapy

## Abstract

**Purpose:**

The aim of this study was to evaluate a program for implementing of the PECS in children with non-verbal ASD or with minimal verbalization in a school clinic belonging to the Unified Health System - SUS.

**Methods:**

This is a longitudinal study. The sample consisted of 22 children with nonverbal ASD or with minimal verbalization; 17 boys and 5 girls, aged 6 to 12 years old. The program consisted of 24 sessions of individual speech language therapy with the presence of the family member and followed the six phases originally proposed by the PECS Training Manual.

**Results:**

All children reached the first three phases. About 82% reached phase IV; 64% phase V and 19% phase VI. Family adherence was 96%.

**Conclusion:**

It was possible to test a PECS implementation program in 24 sessions and verify that children were able to achieve phases of discrimination and sentence construction, besides demonstrating gain in their lexical repertoire and reduction of non-adaptative behaviors.

## INTRODUCTION

Verbal and nonverbal communication disabilities were always considered cardinal aspects for diagnosing autism spectrum disorder (ASD). Current clinical evidence in ASD shows inability to initiate, sustain, and respond to environmental social and communicative demands^(1-3)^.

From an early age, these children’s language and communication precursors point to a deviating and atypical path. That is, nonverbal signs such as deviant gaze, joint attention, and gesture use are hugely impacted and follow a different pattern regarding time, speed of acquisition, and functional use^(2-5)^. Their inability in integrating information with context and meaning, their lack of harmony and synchrony in interpersonal relationships, and their lack of empathy severely compromise these children’s communicative performance and social reciprocity^(2-5)^.

Besides these disabilities, some people with ASD are unable to communicate using speech^(3-5)^. Therefore, they are individuals who benefit from the use of an alternative and augmentative communication system (extended or supplemental) that enables communicative exchange.

The high demand for intervention, especially in the Brazilian public health network, urge for agile and effective approaches that foster the development and adaptation of people with ASD. The Picture Exchange Communication System (PECS) is one of the world's most widely used communication intervention for nonverbal children with autism^(3,5,6)^. It is based on the analysis of applied behavior and consists of a vocabulary repertoire represented by images selected for each user^(5,6)^.

Experienced speech therapists at PECS conduct training in six phases, briefly described below: In phase I (physical exchange: how to communicate), children are encouraged to use cards to request/show their desire for an item that is attractive to them^(5,6)^. Phase II (distance and persistence) aims for children to effectively understand the importance of using cards and keep using them in any communicative situation^5.6^. In phase III (discrimination of pictures), children are encouraged to select a target picture from several options. They must discriminate the cards and deliver the most suitable to each situation to the communication partner. At this phase, children can demonstrate their intentionality by making autonomous choices from reinforcement^(5,6)^. In phase IV (sentence structure), children learn to build sentences using cards with action verbs (e.g. I want) and object attributes (e.g., color, size). This phase considerably expands children functional vocabulary. In phase V (responding to “What do you want?”), children are encouraged to answer the question “What do you want?” using simple sentences with cards^(5,6)^. In phase VI (commenting), children answer questions such as “What do you see?”; “What are you listening to?” “What is this?”, besides spontaneously asking and commenting situations/events using simple sentences with cards^(5,6)^.

Several studies highlight the three initial phases as primordial for a successful implementation of the system, as they boost the ability to discriminate pictures, providing independency and autonomy for children to make their own choices and adhere to the system^(3,5-10)^.

Clinical practice has shown that PECS plays a role in improving verbal comprehension by adding visual and contextual cues to verbal information and, in some cases, it even increases verbal production. However, PECS implementation must be individually evaluated, ensuring all interlocutors engagement^(3-10)^.

Based in our experience from weekly sessions in a school clinic from the Brazilian Unified Health System (SUS), the routine use of cards can only be guaranteed by parents’ engagement in this process, and family empowerment over PECS operation mechanisms is fundamental^(5,7,8)^.

Besides that, we acknowledge the scarce number of multidisciplinary services with qualified and trained professionals to implement this system in Brazil, this study aimed to test a PECS implementation program for nonverbal children with ASD at a school clinic from the Brazilian Unified Health System (SUS).

The hypothesis of this study is that the period stipulated for the implementation of the program will be sufficient for the children to reach the phases of discrimination of figures and construction of sentences, demonstrating appropriation of the system.

## METHOD

**Study design:** This is a longitudinal study.

All parents or guardians were aware of this study methodological procedures and signed the informed consent form approved by the Research Ethics Committee of the Institution (Opinion No. 0809/2018).

**Sample:** A convenience sample of 22 children was analyzed: 17 boys (77%) and five girls (23%), aged between six and 12 years, diagnosed with ASD by a multidisciplinary team according to DSM-5^(1)^ diagnostic criteria and attended at the Center for Speech-Language Hearing Research of Children and Adolescents in Autism Spectrum Disorder - NIFLINC-TEA of the Department of Speech-Language Hearing Science of the UNIFESP, between March 2016 and March 2019.

Regarding communication profile, 86% of children (n=19) presented nonverbal production (babbling and/or vocalizations). Three children (14%) presented minimum verbal production (isolated or juxtaposed words, without verbs).

All children were regularly enrolled in regular schools (due to the Brazilian inclusive education policy) for 45 months on average (SD=21.9) and had been previously exposed to speech intervention in different healthcare services for at least six months to ensure that their communication profile was characterized as nonverbal or minimally-verbal.

No child had been previously exposed to the alternative and augmentative (augmented and supplemental) communication systems.

Regarding families’ socioeconomic status, nine (41%) belonged to classes A/B (upper) and 13 (59%) to classes C/D (lower-middle), according to the Brazilian Association of Research Companies (ABEP) socioeconomic classification^(11)^.

Mothers were 41 years and five months old on average (SD=7.9), and thirteen of them completed higher education (60%) and nine (40%) finished high school.

ASD diagnosis, age group, lack of verbal communication or minimal verbalization, children’s educational link, and family’s availability to participate in speech therapy sessions with a 75% minimum adherence were considered as inclusion criteria.

Children presenting neurological alterations (structural and/or functional involvement of the Central Nervous System), malformations and/or known genetic syndromes, and physical, auditory/visual and/or motor impairments were excluded from the study.

### Procedures

All children were clinically evaluated by a multidisciplinary team composed of child and adolescent psychiatrist, neuropsychologists, and speech therapists. The following instruments were used for collecting data:

- Wechsler Intelligence Scale for Children (WISC III)^(12)^: measures intellectual quotient.
- The Vineland Adaptive Behavior Scale (VABS)^(13)^: questionnaire that investigates social adaptation skills.
- The Autism Behavior Checklist^(14)^: a list of 57 non-adaptive behaviors divided into five categories - sensory, body and object use, relating, language, and social and self-help skills - that measures the degree of probability of ASD.
- The Expressive Vocabulary Test^(15)^: evaluates the lexical repertoire by naming figures grouped by semantic classes.
- The Receptive Vocabulary Test^(15)^: evaluates the receptive vocabulary by identification of figures. In this study, it was applied in its short form.

### The PECS Implementation Program

The program consisted of 24 individual sessions of speech therapy for 45 minutes held weekly with the presence of a family member. All speech-language pathologists were trained and certified professionals in PECS^(5,6)^. As proposed in the PECS Training Manual, all sessions were videotaped to record children's behaviors in the progress monitoring protocols for each phase^(6)^. Records were performed by researchers unrelated to children’s direct care.

Parents actively participated in the entire program, learning how to manage the system in each phase and develop their own materials (example: selecting images, assembling cards). Parents were also encouraged to record everyday situations in the domestic setting so that researchers could certify that they were using the system properly, as well as adapting the setting in each phase of the program (performer’s skills), as proposed by the PECS Training Manual^(6)^. Families engagement to the program was measured by sessions attendance.

After the 24 sessions, children were re-evaluated with some instruments employed in the initial evaluation (ABC, Expressive and Receptive Vocabulary Tests).

**Statistical method:** Initially, descriptive analyses of all variables (age, child´s schooling, intellectual quotient, Vineland, ABC and Vocabulary Test) were performed. Response frequencies for each category were presented when variables were nominal or ordinal. When numerical, measures of central tendency (means and/or medians) and dispersion (standard deviation) were presented. To measure changes resulting from the intervention, measurements obtained at the beginning of the study were compared to those obtained after the 24 sessions using the Wilcoxon Test.

## RESULTS

Table 1 shows the description of the sample.

**Table 1:** Sample Characterization Data

	**Age Child (years)**	**Child Education (months)**	**IQ**	**Vineland**
**Average**	7.2	43.5	50.5	28.7
**Median**	7.1	36.0	49.5	32.0
**SD**	2.1	21.9	9.4	9.2
**N**	22	22	22	22

**Caption:** SD = Standard deviation; N = Number of children

**Figure 1:**
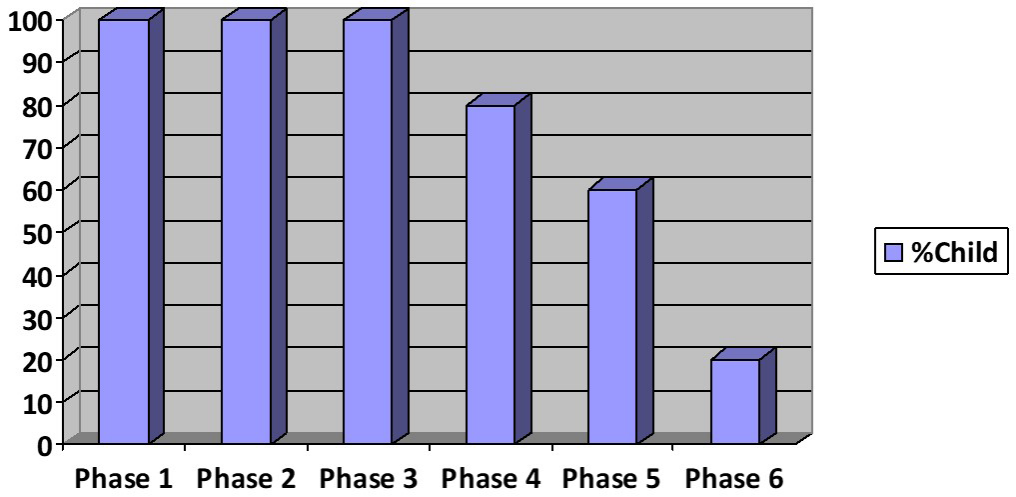
Children’s performance by PECS phases

Figure 1 shows the performance of the children in each phase of the PECS

Table 2 shows the comparative analysis of the ABC and Vocabulary Tests indices in the two moments of the study.

**Table 2:** Total scores and scores by area of ABC and Vocabulary Tests in the two moments

		**Pre**	**Post**	**T-Test or Wilcoxon Test (p)**	**Results**
	Average	13.5	13.2		
**ABC - SE**	Median	14.5	10.0	0.003*	Pre > Post
	SD	4.6	19.6		
	N	22	22		
	Average	22.6	17.5		
**ABC - RE**	Median	22.0	18.5	<0.001*	Pre > Post
	SD	6.7	6.1		
	N	20	20		
	Average	20.4	16.8		
**ABC - BO**	Median	20.0	17.0	0.051	Pre = Post
	SD	8.0	7.4		
	N	22	22		
	Average	13.7	11.5		
**ABC - LG**	Median	13.5	9.5	0.155	Pre = Post
	SD	4.8	5.6		
	N	22	22		
	Average	16.8	10.8		
**ABC - SP**	Median	18.5	10.0	<0.001*	Pre > Post
	SD	4.0	5.0		
	N	22	22		
	Average	87.0	65.1		
**ABC - Total**	Median	86.5	61.0	<0.001*	Pre > Post
	SD	16.5	14.8		
	N	22	22		
	Average	2.4	3.8		
**EXP VOC**	Median	.0	.0	<0.018*	Pre < Post
	SD	8.4	9.2		
	N	22	22		
	Average	2.8	7.0		
**REC VOC**	Median	.0	.0	<0.005*	Pre < Post
	SD	7.7	12.0		
	N	22	22		

(*) statistical significance

**Caption:** ABC = Autism Behavior Checklist; SE = Sensory; RE = Relational; BO = Body and Object Use; LG = Language; SP = Social-Personal; EXP VOC = Expressive Vocabulary; REC VOC = Receptive Vocabulary; Paired t-test or Wilcoxon Test; SD = Standard deviation; N = Number of children

Families’ adherence to sessions was 96%, showing a high engagement to the PECS implementation program in 24 sessions.

## DISCUSSION

Regarding sample characterization, 22 children were attended: 17 boys and five girls. This 4:1 ratio of boys to girls is recurrent and described in epidemiological studies^(1-3)^.

Participants age ranged from six to 12 years, and mean age was 7 years and 2 months (SD=2.1). Although most studies with PECS were conducted with participants aged between five and seven years, some comprised children aged above or below, proving that learning is unrelated to children’ age and all can benefit from this alternative or augmentative communication system^(4-10,16-20)^. In this study, we selected children older than six years who had previously received speech therapy for at least six months, so that the sample communication profile was characterized as nonverbal or minimally-verbal, making it more homogeneous.

As for maternal education, complete higher education (59%) was more predominant than complete high school (41%). These data are very promising, since the literature agrees that maternal education level is a protective factor for child development, as it fosters the understanding of the importance in identifying and treating language impairment in children. In our study, maternal education may have positively influenced the implementation and management of the alternative and augmentative communication system^(4,5,7,8,16-21)^.

The socioeconomic status distribution showed a predominance of families from the C/D classes (lower-middle), equal to 60%, in relation to A/B classes (upper), equal to 40%, indicating a certain sample representativeness in all social classes^(11)^.

Regarding children’s cognitive profile, intelligence quotients distribution was concentrated within the lower range. Some studies with PECS showed that the cognitive ability of children with ASD did not directly interfere during system^(6-10)^.

The Vineland Adaptive Behavior Scale^(15)^ found the predominance of adaptive impairment, confirming that ASD causes disabilities to social development and communicative capacity, as well as the inability to integrate information, compromising the social adaptation of people affected by this condition^(1-5,16-20)^.

### The PECS Implementation Program

By analyzing children’s performance throughout the 24 sessions, we observed that all children were able to discriminate, select, and deliver the target card to the communication partner intentionally and autonomously. Therefore, children had no difficulty in reaching the first three phases of the system.

About 82% of children reached the next phase (phase IV) and started building sentences using cards with action verb and perceptual attributes, presenting a significant increase in their lexical repertoire.

About 64% of children reached phase V, being capable of answering questions such as “what do you want?” using cards^(6)^.

Only 19% of the sample reached phase VI (commenting). Such decrease in performance between phases V and VI is probably related to the task complexity and children’s limitation in understanding and performing the required steps in each of these phases^(6-8,17-24)^. Although less relevant, the intended duration for program implementation (24 sessions) may also have contributed to these results.

The comparative analysis between the two moments of the study showed a tendency to reduce non-adaptive behaviors in all areas of the ABC, with statistical significance in the Sensory (p=0.003), Relating (p=0.001) and Self-help skills (0.001) areas, as well as in the total values (p=0.001). This reduction in atypical behaviors, observed by mothers, confirmed PECS positive impact by enabling more efficient communicative exchanges and improving the quality of social interactions. Even in areas with no statistical significance - Body and Object Use (p=0.051) and Language (p=0.155) - the indices showed a downward trend in the second moment of the study. By adding visual cues to auditory-verbal information, PECS improved verbal comprehension and positively impacted communicative exchanges and social engagement^(7-10,20-24)^.

We also verified a significant increase in responses in both expressive (p=0.018) and receptive (p=0.005) vocabulary test. Communicative performance improvement also influenced social adaptation, probably because PECS enabled a greater attentiveness to social and communication cues among its users. Other studies also reported a positive effect of PECS in increasing verbalization^(7-10,20-24)^.

Families’ adherence to sessions was 96%, showing a high engagement to the PECS implementation program in 24 sessions. These are very positive results, as we observed that mothers were attentive to all guidelines and were able to report their doubts and difficulties regarding the use of PECS within the domestic setting. Although the evaluation for children to commence a new phase does not intrinsically depends on their parents executing the tasks^(4-8)^. We believe that family engagement and involvement were essential to ensure children's adherence to the system.

Another relevant point is the high family support and children’s highly satisfactory performance in a program of only 24 sessions, for 45 minutes, and held weekly. These data suggest that the program can be successfully reproduced in various regions of the country provided that professionals are duly trained, and family engagement is ensured, as only these factors will guarantee the amount of teaching hours and children’s adherence to the system.

Encouraging the implementation of an alternative and augmentative communication system for nonverbal or verbal children with ASD goes far beyond children's training, requiring moments of joint reception, explanation, and reflection between families and therapists^(3-8,20-24)^. Brazil is a country of continental proportions with great social, economic, and cultural inequalities^(21)^. Therefore, rethinking care models that promote families’ empowerment and access to treatment as equally as possible will only succeed if everyone is engaged and aware of their participation in the process of building this service model.

### Study limitations

In delineating an implementation program for PECS in 24 sessions, we sought to test a model focused on people with ASD that could be reproduced in public health services to reduce queues and improve families’ access to speech therapy. However, the stipulated implementation time should be considered a limitation of the study. At the end of the program, all children assisted were referred to other services to give continuity to speech therapy, ideally closer to their homes. We were available to collaborate with the respective clinic schools and therapeutic teams during the entire assisting period.

The sample communication and age profiles were carefully designed to avoid disparities in the results, especially of vocabulary tests. We suggest further studies to be conducted with larger samples and more heterogeneous regarding age group and linguistic level, as well as with a longer exposure time to the system. Finally, we acknowledge that randomized double-blind trials are strongly recommended for testing the efficacy of PECS use.

## CONCLUSIONS

It was possible to evaluate a PECS implementation program in 24 sessions and observe that the engagement of families was very important for children to take ownership of the system. In a complementary way, it is believed that the program can be successfully reproduced since it can be verified that the children could reach phases of discrimination and sentence construction, in addition to demonstrating gain in their lexical repertoire and reduction in non-adaptative behaviors.

## References

[1] American Psychiatric Association (2014). Manual Diagnóstico e Estatístico de Transtornos Mentais - DSM-5.

[2] Klin A, Jones W (2018). An agenda for 21st century neurodevelopmental medicine: lessons from autism. Rev Neurol.

[3] Tamanaha AC, Perissinoto J (2019). Transtornos do Espectro do Autismo - implementando estratégias para a comunicação..

[4] Moretto G, Ishihara MK, Ribeiro M, Caetano SC, Perissinoto J, Tamanaha AC (2020). Interference of the communicative profile of children with Autism Spectrum Disorders upon their mother’s quality of life. CoDAS.

[5] Santos PA, Bordini D, Scattolin M, Asevedo GRDC, Caetano SC, Paula CS (2021). The impact of the implementation of PECS on understanding instruction in children with Autism Spectrum Disorders. CoDAS.

[6] Bondy A, Frost L (2009). Manual de treinamento do sistema de comunicação por Troca de Figuras..

[7] Ferreira C, Caetano SC, Perissinoto J, Tamanaha AC (2022). Repercussão da implementação do Picture Exchange Communication System - PECS no índice de sobrecarga de mães de crianças com Transtorno do Espectro do Autismo. CoDAS.

[8] Olivatti DFO, Sugahara MK, Camilo S, Perissinoto J, Tamanaha AC (2021). Relevância do engajamento familiar na implementação do Picture Exchange Communication System - PECS em crianças com Transtorno do Espectro do Autismo. Rev CEFAC.

[9] Jurgens A, Anderson A, Moore DW (2019). Maintenance and generalization of skills acquired through PECS training: a long-term follow-up. Dev Neurorehabil.

[10] Pereira ET, Montenegro ACA, Rosal AGC, Walter CCF (2020). Augmentative and alternative communication on Autism Spectrum Disorder: impacts on communication. CoDAS.

[11] ABEP: Associação Brasileira de Empresas de Pesquisa (2018). Critério de Classificação Econômica Brasil.

[12] Weschler D (2002). WISC III Escala de inteligência para crianças..

[13] Sparrow SS, Balla D, Cicchetti D (1984). Vineland adaptative behavior scales. Expanded edition.

[14]  Marteleto MRF, Pedromônico MRM (2005). Validity of Autism Behavior Checklist (ABC): preliminary study. Rev Bras Psiquiatr.

[15] Capovilla FC, Negrão VD, Damásio M (2011). Teste de vocabulário auditivo e teste de vocabulário expressivo.

[16] Ribeiro SH, Paula CS, Bordini D, Mari JJ, Caetano SC (2017). Barriers to early identification of Autism in Brazil. Rev Bras Psiquiatr.

[17] Donato C, Spencer E, Arthur-Kelly M (2018). A critical synthesis of barriers and facilitations to the use of AAC by children with ASD and their communication partners. Augment Altern Commun.

[18] Sievers SB, Trembath D, Westerveld M (2018). A systematic review of predictors, moderators and mediators of augmentative and alternative communication outcomes for children with ASD. Augment Altern Commun.

[19] White EN, Ayres KM, Snyder SK, Cagliani RR, Ledford JR (2021). Augmentative and alternative communication and speech production for individuals with ASD: a systematic review. J Autism Dev Disord.

[20] Klin A, Micheletti M, Klalman CI, Schultz S, Constantino JN, Jones W (2020). Affording autism in early brain development re-definition. Dev Psychopathol.

[21] Micheletti M, McCracken C, Constantino J, Mandell D, Jones W, Klin A (2020). Outcomes of 24 to 36 months-old children with ASD vary by ascertainment strategy: a systematic review and meta-analysis. J Child Psychol Psychiatry.

[22] Lai MC, Anagnostou E, Wiznitzer M, Alisson C, Baron Cohen S (2020). Evidence-based support for autistic people across the lifespan: maximizing potential, minimizing barriers, and optimizing the person-environment fit. Lancet Neurol.

[23] Brignell A, Chenausky KV, Song H, Zhu J, Suo C, Morgan AT (2018). Communication intervention for Autism Spectrum Disorder in minimally verbal children. Cochrane Database Syst Rev.

[24] Gilroy SP, Leader G, McCleery JP (2018). A pilot community-based randomized comparison of speech generating devices and the PECS for children diagnosed with Autism Spectrum Disorder. Autism Res.

