# Supplementary material for: Picture Exchange Communication System (PECS) Implementation Program for children with autism spectrum disorder
Source: Codas. 2023 Sep 18;35(4):e20210305. doi: 10.1590/2317-1782/20232021305en (PMC10546919; doi:10.1590/2317-1782/20232021305en)
Supplement: Translation

# Translation: Programa de Implementação do *Picture Exchange Communication System* (PECS) para crianças com Transtorno do Espectro do Autismo

**DOI:** 10.1590/2317-1782/20232021305pt

**Authors:** Ana Carina Tamanaha, Dayane Oliveira Felicio Olivatti, Simoni Camilo da Silva, Soraia Cunha Peixoto Vieira, Jacy Perissinoto

**Affiliations:** 1 Departamento de Fonoaudiologia, Universidade Federal de São Paulo - UNIFESP - São Paulo (SP), Brasil.; 2 Instituto de Educação de Londres - Belo Horizonte (MG), Brasil.

**Keywords:** Autismo, Comunicação, Linguagem, Auxiliares de Comunicação para Pessoas com Deficiência, Fonoaudiologia

## Abstract

**Objetivo:**

O objetivo deste estudo foi avaliar um programa de implementação do PECS em crianças com TEA em clínica-escola pertencente ao Sistema Único de Saúde - SUS.

**Método:**

Trata-se de estudo longitudinal. Foram atendidas 22 crianças com TEA não falantes ou com verbalização mínima; sendo 17 do gênero masculino e 5 do feminino, na faixa etária de 6 a 12 anos. O programa foi constituído por 24 sessões de terapia fonoaudiológica individual com a presença do familiar e obedeceu às seis fases propostas originalmente pelo Manual de Treinamento do PECS.

**Resultados:**

Todas as crianças atingiram apropriadamente as três primeiras fases. A fase IV foi alcançada por 82% delas; a fase V por 64%; e a fase VI por cerca de 19% da amostra. O índice de adesão das famílias foi de 96%.

**Conclusão:**

Foi possível testar um programa de implementação do PECS em 24 sessões e verificar que as crianças puderam atingir fases de discriminação e construção de frases, além de demonstrarem ganho em seu repertório lexical e diminuição das atipias comportamentais.

## INTRODUÇÃO

As inabilidades da comunicação verbal e não verbal sempre foram consideradas aspectos cardinais para o diagnóstico do Transtorno do Espectro do Autismo (TEA). As evidências clínicas atuais do TEA mostram inabilidades tanto para iniciar, sustentar, como para responder às demandas sociais e comunicativas do ambiente^(1-3)^.

Os precursores da linguagem e da comunicação apontam, desde muito cedo, para um trajeto desviante e atípico. Ou seja, sinais não verbais como o direcionamento do olhar, o compartilhamento de atenção e o uso de gestos, sofrem forte impacto e seguem um curso diferente em relação ao tempo, velocidade de aquisição e uso funcional^(2-5)^. A inabilidade em integrar informações, com contexto e significado, a falta de sintonia e de sincronia nas relações interpessoais e a ausência de empatia comprometem demasiadamente o desempenho comunicativo e a própria reciprocidade social^(2-5)^.

Soma-se a esses prejuízos o fato de um contingente de pessoas com TEA não ser capaz de se comunicar por meio da fala^(3-5)^. Portanto, são indivíduos que se beneficiam do uso de sistema de comunicação alternativo e aumentativo (ampliado ou suplementar) e que viabilize a troca comunicativa.

A grande demanda para a intervenção, especialmente na rede pública de saúde brasileira, pede a busca de abordagens ágeis e eficazes que impulsionem o desenvolvimento e adaptação das pessoas com TEA. O PECS (*Picture Exchange Communication System*) é um dos sistemas de comunicação utilizados para pessoas do espectro autistico com severo comprometimento de comunicação^(3,5,6)^. Ele baseia-se na análise do comportamento aplicada e é constituído por um repertório de vocabulário representado por imagens selecionado para cada usuário^(5,6)^.

Profissionais com experiência e certificados implementam o PECS em seis fases: na fase I (Troca física: como comunicar) o usuário aprende a usar os cartões para indicar o que deseja^(5,6)^. Na fase II (Distância e persistência): persiste-se no uso dos cartões em diferentes contextos^(5,6)^. Na fase III (Discriminação de figuras) a ênfase é na discriminação visual e seleção adequada de diversos cartões. Na fase IV (Estrutura da sentença) aprende-se a construção de frases utilizando-se cartões relativos a verbos de ação (ex: querer) e atributos dos objetos (ex: cor)^(5,6)^. Na fase V (Responder ao que você quer?) o usuário é incentivado a responder à pergunta “O que você quer?”^(5,6)^. E na fase VI (Comentar) aprende-se a criar comentários tais como “eu estou assistindo...”^(5,6)^.

Diversos estudos têm apontado as três fases iniciais como as primordiais para uma implementação bem-sucedida do sistema, uma vez que promovem a capacidade de discriminação das figuras, tornando o usuário independente e autônomo em suas escolhas e em sua apropriação do sistema^(3,5-10)^.

Além disso, o uso do PECS pode potencializar a compreensão verbal à medida que agrega pista visual à informação auditivo-verbal e em alguns casos, promove o aumento da verbalização^(3-6)^. No entanto, é fundamental que a sua implementação seja criteriosa, e a participação efetiva de todos os interlocutores seja garantida^(3-10)^.

Na experiência em clínica escola inserida no Sistema Único de Saúde (SUS) brasileiro, tem-se identificado a partir de sessões semanais, que o uso rotineiro dos cartões só pode ser garantido a partir do engajamento dos pais neste processo e para tanto é fundamental o empoderamento familiar sobre os mecanismos de funcionamento do PECS^(5,7,8)^.

Cientes de que são poucos os serviços multidisciplinares com profissionais capacitados e treinados para implementação deste sistema no Brasil, o objetivo deste estudo foi avaliar um programa de implementação do PECS em crianças com TEA em clínica-escola pertencente ao Sistema Único de Saúde - SUS.

A hipótese deste estudo é a de que o período estipulado para implementação do programa será suficiente para que as crianças atinjam as fases de discriminação de figuras e construção de frases, demonstrando apropriação do sistema.

## MÉTODO

**Desenho do estudo:** Trata-se de um estudo longitudinal.

Todos os pais ou responsáveis assinaram o Termo de Consentimento Livre e Esclarecido aprovado pelo Comitê de Ética em Pesquisa da Instituição (Parecer N° 0809/2018), consentindo com os procedimentos metodológicos do estudo.

**Amostra:** Foi constituída uma amostra de conveniência com 22 crianças, sendo dezessete do gênero masculino (77%) e cinco, do feminino (23%); com idade variando entre 6 a 12 anos, diagnosticadas com TEA por equipe multidisciplinar segundo os critérios diagnósticos do DSM 5^(1)^, e atendidas no Núcleo de Investigação Fonoaudiológica de Linguagem da Criança e do Adolescente no Transtorno do Espectro do Autismo - NIFLINC-TEA do Departamento de Fonoaudiologia da UNIFESP, no período compreendido entre março de 2016 a março de 2019.

Em relação ao meio comunicativo 86% das crianças (n=19) emitiram apenas balbucio e/ou vocalizações e foram classificadas como não-falantes. Três crianças (14%) produziram verbalização mínima (palavras isoladas ou justaposição, com ausência de uso de verbos).

O tempo médio de escolaridade das crianças foi de quarenta e cinco meses (DP=21,9). Todas as crianças estavam matriculadas em escolas de ensino regular (59% público e 41%, privado) e já haviam sido assistidas anteriormente em terapia fonoaudiológica em diferentes serviços, no mínimo por seis meses, para a garantia de que o meio comunicativo fosse classificado como não verbal ou por verbalização mínima. Nenhuma criança havia sido exposta previamente aos sistemas de comunicação alternativo e aumentativo (ampliado e suplementar).

A classificação socioeconômica da ABEP^(11)^ indicou que nove famílias (41%) pertenciam às classes A/B (altas) e treze (59%) às classes C/D (média-baixa).

As mães tinham, em média, 41 anos e 5 meses (DP=7,9); e treze delas haviam completado ensino superior (59%) e nove (41%) ensino médio.

Considerou-se como critérios de inclusão: diagnóstico multidisciplinar de TEA, a faixa etária, comprometimento severo de comunicação e a frequência (adesão) mínima de 75% às sessões do programa.

Como critérios de exclusão foi considerada: a presença de alterações neurológicas (acometimento estrutural e/ou funcional do Sistema Nervoso Central); malformações e/ou síndromes genéticas conhecidas; e déficit físico, auditivo, visual e/ou motor.

### Procedimentos

Foram aplicados os seguintes instrumentos no período inicial de avaliação fonoaudiológica:

- Escala de Inteligência Weschler - WISC III^(12)^: mensura o quociente intelectual.
- Escala Vineland de Comportamento Adaptativo^(13)^: questionário que investiga as habilidades de adaptação social.
- Autism Behavior Checklist^(14)^: listagem de comportamentos atípicos agrupados nas áreas: sensorial, uso do corpo e objeto, relacional, linguagem e pessoal-social, e que mensura o grau de probabilidade de TEA.
- Teste de Vocabulário Expressivo^(15)^: avalia o repertório lexical por meio de nomeação de figuras agrupadas por classes semânticas.
- Teste de Vocabulário Auditivo (forma abreviada)^(15)^: avalia o vocabulário receptivo por meio da identificação de figuras.

### O Programa de Implementação do PECS

Foi constituído por 24 sessões de terapia fonoaudiológica individual com a presença do familiar. Cada sessão teve duração de 45 minutos e foi realizada semanalmente. Todos os fonoaudiológicos envolvidos eram profissionais treinados e certificados no PECS^(5,6)^. Todas as sessões foram filmadas para que os comportamentos das crianças fossem registrados nos protocolos de monitoramento dos progressos em cada fase, conforme proposto no Manual de Treinamento do PECS^(6)^. Os registros foram feitos por pesquisadores que não estiveram envolvidos no atendimento direto das crianças.

Os pais participaram ativamente de todo programa: aprendendo o manejo do sistema em cada fase e construindo seus próprios materiais (exemplo: selecionar imagens, montar cartões). Além disso, foram incentivados a registrar por meio de filmagens, situações cotidianas em ambiente doméstico para que os pesquisadores pudessem se certificar do uso apropriado do sistema, bem como da preparação do ambiente por eles, em cada fase do programa (habilidades do executor) conforme proposto pelo Manual de Treinamento do PECS^(6)^. Além disso, o engajamento (adesão) das famílias ao programa foi mensurado pela frequência às sessões.

Após as 24 sessões realizadas as crianças foram reavaliadas com parte dos instrumentos (*Autism Behavior Checklist* e Teste de Vocabulário Expressivo e Auditivo) utilizados na etapa inicial de avaliação.

**Método estatístico**: Foram realizadas análises descritivas de todas as variáveis do estudo (idade, escolaridade da criança, quociente intelectual, índices da Vineland, do ABC e dos Testes de Vocabulário). Quando as variáveis eram numéricas foram apresentadas medidas de tendência central (médias e/ou medianas) e de dispersão (desvio padrão). Para mensuração de mudanças ocorridas como resultado da intervenção, as medidas iniciais e finais foram comparadas por meio do Teste Wilcoxon.

## RESULTADOS

Na Tabela 1 tem-se a descrição da amostra.

**Tabela 1:** Dados descritivos da amostra

	**Idade criança (anos)**	**Escolaridade criança (meses)**	**QI**	**Vineland**
**Média**	7,2	43,5	50,5	28,7
**Mediana**	7,1	36,0	49,5	32,0
**DP**	2,1	21,9	9,4	9,2
**N**	22	22	22	22

**Legenda:** DP = Desvio padrão; N = número de crianças

Na Figura 1 observa-se o desempenho das crianças em cada fase do PECS.

**Figura 1:** Desempenho das crianças por fases do PECS

Na Tabela 2 tem-se a análise comparativa dos índices do ABC e dos Testes de Vocabulário nos dois momentos do estudo.

**Tabela 2:** Escores Totais e por áreas do ABC e dos Testes de Vocabulário nos dois momentos do estudo

		**Pre**	**Pós**	**T-Teste ou Teste Wilcoxon (p)**	**Resultados**
	Média	13,5	13,2		
**ABC - SE**	Mediana	14,5	10,0	0,003*	Pré > Pós
	DP	4,6	19,6		
	N	22	22		
	Média	22,6	17,5		
**ABC - RE**	Mediana	22,0	18,5	<0,001*	Pré > Pós
	DP	6,7	6,1		
	N	22	22		
	Média	20,4	16,8		
**ABC - CO**	Mediana	20,0	17,0	0,051	Pré = Pós
	DP	8,0	7,4		
	N	22	22		
	Média	13,7	11,5		
**ABC - LGG**	Mediana	13,5	9,5	0,155	Pré = Pós
	DP	4,8	5,6		
	N	22	22		
	Média	16,8	10,8		
**ABC - OS**	Mediana	18,5	10,0	<0,001*	Pré > Pós
	DP	4,0	5,0		
	N	22	22		
	Média	87,0	65,1		
**ABC - Total**	Mediana	86,5	61,0	<0,001*	Pré > Pós
	DP	16,5	14,8		
	N	22	22		
	Média	2,4	3,8		
**VOC EXP**	Mediana	,0	,0	<0,018*	Pré < Pós
	DP	8,4	9,2		
	N	22	22		
	Média	2,8	7,0		
**VOC AUD**	Mediana	,0	,0	<0,005*	Pré < Pós
	DP	7,7	12,0		
	N	22	22		

(*) significância estatística

**Legenda:** ABC = Autism Behavior Checklist, SE = Sensorial, RE = Relacional, CO = Uso do Corpo e Objeto, LGG = Linguagem, PS = Pessoal-Social, VOC EXP = Vocabulário Expressivo, VOC AUD = Vocabulário Auditivo, Test T-pareado ou Wilcoxon, DP = Desvio padrão, N = número de crianças

A frequência média das famílias às sessões foi de 96%, mostrando alto engajamento (adesão) ao programa de implementação do PECS em 24 sessões.

## DISCUSSÃO

Foram atendidas vinte e duas crianças, sendo dezessete meninos e cinco meninas. Essa proporção maior do gênero masculino em relação ao feminino corrobora as descrições dos estudos epidemiológicos no TEA^(1-3)^.

Em relação à faixa etária das crianças a média de idade foi de 7 anos e 2 meses (DP=2,1). Embora grande parte das pesquisas sobre o uso do PECS tenham sido conduzidas com crianças nesta mesma faixa etária, os pesquisadores foram unanimes em apontar que a variável idade não interferiu diretamente na aprendizagem do sistema^(4-10,16-20)^. Particularmente neste estudo optou-se a priori selecionar crianças acima de seis anos de idade e que já tivessem sido expostas à terapia fonoaudiológica por um período mínimo de seis meses para que o meio comunicativo da amostra fosse caracterizado como não-verbal ou por verbalização mínima, tornando a amostra mais homogênea.

Quanto à escolaridade materna, pondera-se que o predomínio de nível superior completo (59%) seguido de nível médio (41%) pode ter influenciado de forma positiva a implementação do programa à medida que o acesso das mães à educação, é fator de proteção ao desenvolvimento da criança, portanto, pode ter potencializado o aprendizado e o engajamento materno ao sistema^(4,5,7,8,16-21)^.

Houve enquadramento de cerca de 60% das famílias nas classes C/D (média-baixa) e 40% nas classes A/B (alta), de acordo com a classificação da ABEP^(11)^.

Em relação ao perfil cognitivo os índices de quociente intelectual concentraram-se na faixa inferior. Pesquisas conduzidas com o PECS mostraram que o potencial cognitivo dos indivíduos não interferiu na aprendizagem do sistema, mesmo tendo o usuário um grau severo de comprometimento intelectual^(6-10)^.

Na Escala Vineland de Comportamento Adaptativo^(13)^ as respostas obtidas indicaram predomínio de classificação de nível deficitário, confirmando que no TEA existem prejuízos nos desenvolvimentos social e comunicativo, que impactam profundamente a adaptação social das pessoas acometidas por essa condição^(1-5,16-20)^.

### O Programa de Implementação do PECS

Durante o período de 24 sessões verificou-se que todas as crianças aprenderam a discriminar e selecionar o cartão alvo e entregá-lo ao parceiro de comunicação de modo intencional e autônomo. Sendo assim, o alcance das primeiras três fases ocorreu em 100% da amostra.

A fase IV foi alcançada por 82% das crianças à medida que elas passaram a construir frases usando cartões de verbos de ação e atributos perceptuais, mostrando aumento expressivo no repertório lexical.

Cerca de 64% das crianças mostraram-se hábeis em atingir a fase V, respondendo a questionamentos tais como: “o que você quer?” utilizando os cartões adequadamente, conforme proposto pelos autores do sistema de comunicação por troca de figuras^(6)^.

Apenas 19% da amostra atingiu a fase VI (Comentários). Essa redução de desempenho entre as duas últimas fases do PECS deve vincular-se à complexidade das ações atribuídas nestas fases e à decorrente limitação da criança em compreendê-las e executá-las^(6-8,17-24)^. Outro fator, embora menos relevante, mas que também pode ter colaborado para esses resultados refere-se ao intervalo de tempo estimado para a implementação do programa (24 sessões).

Na análise comparativa entre os dois momentos do estudo, verificou-se que houve tendência de redução dos comportamentos não adaptativos observada em todas as áreas do ABC. Sendo que houve significância estatística nas áreas Sensorial (p=0,003), Relacional (p=0,001) e Pessoal-Social (0,001), bem como nos valores Totais (p=0,001). Essa diminuição de atipias comportamentais, observada pela perspectiva das mães, confirmou o impacto positivo do uso do PECS, não apenas possibilitando trocas comunicativas mais eficientes, mas também melhorando a própria qualidade das interações sociais. Mesmo nas áreas em que não houve significância estatística: Uso do Corpo e Objeto (p=0,051) e Linguagem (p=0,155), os índices no segundo momento do estudo mostraram tendência de redução. Ao agregar pistas visuais à informação auditivo-verbal, o PECS ampliou a compreensão verbal e impactou positivamente as trocas comunicativas e o engajamento social cotidiano^(7-10,20-24)^.

Observou-se ainda, aumento significativo de respostas tanto na testagem do vocabulário expressivo (p=0,018), quanto auditivo (p=0,005). A melhora no desempenho comunicativo, tanto em seu caráter expressivo quanto receptivo, corroboram o efeito positivo do PECS no aumento de verbalização e sobretudo na compreensão verbal de seus usuários, conforme descrito por diferentes estudos^(7-10,20-24)^.

O engajamento das famílias foi de 96%, em média, mostrando alta adesão ao Programa de Implementação do PECS em 24 sessões. Esses resultados são bastante positivos à medida que pude-se observar que as mães estiveram atentas as orientações fornecidas e puderam relatar suas dúvidas e dificuldades em relação ao uso do PECS em rotina doméstica. E embora a avaliação para passagem pelas fases pelas crianças não dependa intrinsecamente da execução das tarefas pelo adulto familiar^(4-8)^, acredita-se que o engajamento e o envolvimento familiar foram essenciais para garantia da apropriação do sistema pelas crianças.

Outro ponto relevante que deve ser valorizado é o fato de termos alto engajamento (adesão) das famílias e desempenho bastante satisfatório das crianças em um programa de apenas 24 sessões, realizadas em 45 minutos e apenas uma vez por semana. Esses dados nos permitem acreditar que o programa possa ser reproduzido de forma bem-sucedida em várias regiões do país, desde que haja além do treinamento adequado dos profissionais, a garantia do engajamento familiar (adesão), pois só assim garantiremos a quantidade de horas de ensino e apropriação do sistema pelas crianças.

Assim, incentivar a implementação de um sistema de comunicação alternativo e aumentativo para crianças com TEA não falantes ou com verbalização vai muito além do treinamento da criança, são necessários momentos de acolhimento, explicação e reflexão conjuntas entre famílias e terapeutas^(3-8,20-24)^. O Brasil é um país de proporções continentais e com grandes desigualdades sociais, econômicas, culturais^(21)^; portanto repensar modelos de atendimento que possam promover o empoderamento das famílias, o alcance do tratamento de forma mais igualitária possível, são tarefas que só terão êxito se todos estiverem envolvidos e conscientes de sua participação no processo de construção deste modelo de atendimento.

### Limitações do estudo

Ao delinearmos um programa de implementação para o PECS em 24 sessões buscou-se testar um modelo que pudesse ser reproduzido nos serviços de saúde pública direcionados às pessoas com TEA, com intuito de reduzir as filas de espera e potencializar o acesso das famílias ao atendimento fonoaudiológico. No entanto, temos ciência que o tempo de implementação estipulado neste estudo deve ser considerado uma limitação do estudo. Neste sentido, é importante mencionar que todas as crianças assistidas foram redirecionadas para outros serviços para que pudessem dar continuidade ao atendimento fonoaudiológico, idealmente mais próximo de suas residências, ao final do programa. Além disto, durante todo o período em que as crianças e suas famílias foram assistidas estivemos disponíveis para colaborar com as respectivas escolas e equipes terapêuticas.

Os perfis comunicativo e etário da amostra também foram cuidadosamente delineados para evitarmos disparidades nos resultados, especialmente, dos testes de vocabulário. Sugere-se que mais estudos sejam conduzidos com amostras maiores e mais heterogêneas em relação a faixa etária e nível linguístico; e por um tempo mais longo de exposição ao sistema. E por fim, temos ciência que ensaios clínicos randomizados com duplo cego são fortemente recomendados para testagem da eficácia do uso do PECS.

## CONCLUSÕES

Foi possível avaliar um programa de implementação do PECS em 24 sessões e observar que o engajamento das famílias foi muito importante para que as crianças se apropriassem do sistema. De forma complementar, acredita-se que o programa poderá ser reproduzido com êxito uma vez que pode-se verificar que as crianças puderam atingir fases de discriminação e construção de frases, além de demonstrarem ganho em seu repertório lexical e diminuição das atipias comportamentais.
